# First molecular identification and phylogenetic illustration of *Sarcocystis* species infection in Red Sea shortfin mako shark (*Isurus oxyrinchus* Rafinesque, 1810)

**DOI:** 10.1186/s12917-024-03952-w

**Published:** 2024-03-15

**Authors:** Nahla HE. M. Ahmed, Ahmed Ghallab, Mohamed Shaalan, Mahmoud Saied, Eman Sayed Mohammed

**Affiliations:** 1 National Institute of Oceanography and Fisheries, NIOF, Cairo, Egypt; 2 Natural Conservation Sector, Ministry of Environment, Cairo, Egypt; 3 Department of Pathology, Faculty of Veterinary Medicine, Cairo University, Cairo, Egypt; 4 Department of Parasitology, Faculty of Veterinary Medicine, South Valley University, Qena, 83523 Egypt

**Keywords:** Mako Shark, *Sarcocystis* sp., Macroscopic, Molecular, 18S rRNA, Red Sea

## Abstract

**Background:**

members of the genus *Sarcocystis* are intracellular obligate protozoan parasites classified within the phylum Apicomplexa and have an obligate heteroxenous life cycle involving two hosts. A more comprehensive understanding of the prevalence and geographic range of different *Sarcocystis* species in marine ecosystems is needed globally and nationally. Hence, the objective of this study was to document the incidence of *Sarcocystis* infection in sharks within the aquarium ecosystem of Egypt and to identify the species through the characterization of the SSU rDNA gene.

**Methods:**

All organs of the mako shark specimen underwent macroscopic screening to detect the existence of a *Sarcocystis* cyst. Ten cysts were collected from the intestine and processed separately to extract the genomic DNA. The polymerase chain reaction (PCR) was accomplished by amplifying a specific 18S ribosomal RNA (rRNA) gene fragment. Subsequently, the resulting amplicons were subjected to purification and sequencing processes.

**Results:**

Macroscopic examination of the mako shark intestinal wall sample revealed the presence of *Sarcocysti*s cysts of various sizes and shapes, and sequencing of the amplicons from *Sarcocystis* DNA revealed a 100% nucleotide identity with the sequence of *Sarcocystis tenella* recorded from sheep in Iran; The mako shark sequence has been deposited in the GeneBank with the accession number OQ721979. This study presents the first scientific evidence demonstrating the presence of the *Sarcocystis* parasite in sharks, thereby documenting this specific marine species as a novel intermediate host in the Sarcocystis life cycle.

**Conclusions:**

This is the first identification of *Sarcocystis* infection in sharks, and we anticipate it will be an essential study for future screenings and establishing effective management measures for this disease in aquatic ecosystems.

**Supplementary Information:**

The online version contains supplementary material available at 10.1186/s12917-024-03952-w.

## Background

*Sarcocystis* species are cyst-forming intracellular apicomplexans with alternating life cycles involving an intermediate and a final host. Carnivores are the final hosts for some species of this genus. However, *Sarcocystis* parasites can infect a wide range of intermediate hosts, including mammals, birds, reptiles, and aquatic organisms. The final host develops infection by ingesting cysts harboring infective bradyzoites, which are present in the muscle tissue of the intermediate host. Bradyzoites undergo sexual phases within the intestine of the final host, leading to the formation of oocysts/sporocysts, which are subsequently excreted in the feces. Infection of the intermediate host begins with ingestion of sporulated oocysts in feces or water; once sporozoites are released and divide in the gut, they travel to the striated muscles and other organs, where *Sarcocystis* develops [1, 2]. A review of the available literature shows that approximately 150–200 species of *Sarcocystis* have been identified and described in a wide range of hosts, mostly based on parasite morphology [3]. In cattle, 12 *Sarcocystis* spp. have been reported, including *Sarcocystis cruzi, S. hominis, S. hirsuta, S. rommeli, S. heydorni, S. bovifelis, S. bovini, S. sinensis, S. gigantea, S. fusiformis, S. hjorti*, and *S. tenella*. In sheep [2], six *Sarcocystis* species are known, which are *Sarcocystis gigantea, S. medusiformis, S. tenella, S. arieticanis, S. microps*, and *S. mihoensis.* [2, 4]. Moreover, five *Sarcocystis* spp. in camel were identified as *Sarcocystis cameli, S. ippeni, S. camelicanis, S. camelocanis*, and *S. miescheri* [5].

It is worth noting that *Sarcocystis* spp. are among the most prevalent protist parasites that have economic importance; thus, *Sarcocystis* has been observed in many wild and domestic mammals [6]. However, few reports have recorded the occurrence of *Sarcocystis* spp. in marine mammals such as pinnipeds [7, 8], cetaceans [8–10], and sea otters [11, 12].

To the best of our knowledge, no molecular screening strategy has been implemented before to scout the occurrence of *Sarcocystis* infections in sharks. Hence, the present investigation aimed to determine the existence of *Sarcocystis* sp. in shark specimens in the Red Sea, Egypt. The molecular identification of the retrieved *Sarcocystis* specimens was performed using sequence analysis of the 18S rRNA gene.

## Materials and methods

### Study framework

The current study was conducted between July 2018 and October 2022 in Hurghada City, the capital of the Red Sea Governorate of Egypt (coordinate: 27° 15’ 26.57” N 33° 48’ 46.48” E).

### Fish samples preparation

**Fig. 1:**
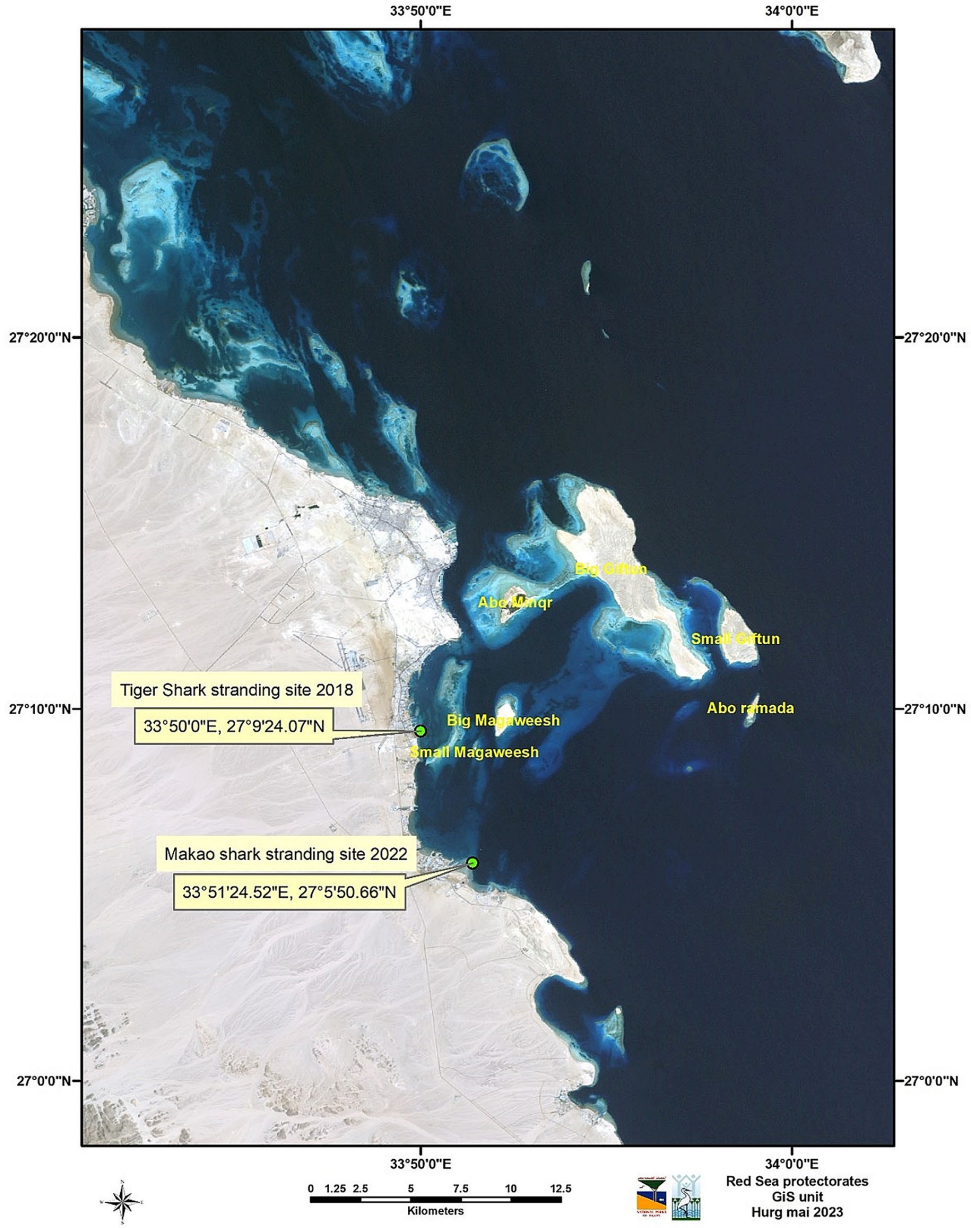
A map showing the sampling locations of both tiger sharks and shortfin mako shark samples. The map was created using ArcGIS software

Sampling was performed on two tiger sharks that were found dead in 2018 (coordinate: 27° 9’24.07” N, 33° 50’ 0” E) and one specimen of mako shark found dead in 2022 (coordinate: 27° 5’50.066” N, 33° 51’ 24.52” E) (Fig. 1). After documenting all external and internal postmortem abnormalities in all organs and identifying the species and sex, the collected shark samples were transmitted to the National Institute of Oceanography and Fisheries, NIOF, Hurghada, Egypt, for necropsy purposes.

### Molecular identification

#### Genomic isolation, purification, and specific gene amplification

Ten macroscopic cysts were collected from the intestinal wall of the shortfin mako shark and preserved in 99% ethanol for molecular identification. The DNA was extracted from each of the ten cysts separately and subjected to purification using the Gene Jet Genomic DNA purification Mini Kit (Thermo Scientific), following the manufacturer’s instructions. The purified DNA sample was stored at -20 °C until PCR analysis. DNA amplifications were carried out using a CreaCon thermal cycler, and a DNA molecular weight ladder 1kbp DNA marker (PeqGold 1Kb, Peqlab, and GMH) was used to estimate the final amplified product length on agarose gels.

A specific set of primers (Sar-F1 5’GCACTTGATGAATTCTGGCA3’; Sar-R1 5’CACCACCCATAGAATCAAG 3’) were supplied from Macrogen (Korea) for the amplification of the 18S rRNA gene of *Sarcocystis* parasite [13]. DreamTaq Green polymerase master mix (Thermo Scientific, USA) was used for gene amplification according to the manufacturer’s guidelines. Briefly, The PCR reaction mixture consisted of 50 ng of genomic DNA extracted from the cyst sample, 50 pmol of each primer, 200 μm of each deoxyribonucleotide triphosphate (dNTP), 5 µl of a 10-fold concentrated PCR buffer (comprising 100 mM Tris-HCI at pH 9, 15 mM MgCl_2_, and 500 mM potassium chloride), and 1.0 U of Taq polymerase in a final volume of 50 µl. The thermal cycler conditions used consisted of a pre-denaturation step at 94 °C for 5 min, followed by 30 cycles of denaturation at 94 °C for 45 s, annealing at 55 °C for 1 min, and extension at 72 °C for 1 min. The last cycle included an elongation step of 7 min at 72 °C.

### Documentation of the PCR products

The PCR product was electrophoresed on a 2% (w/v) Agarose agarose gel (Agarose, universal, peq GOLD, peqlab Germany) in 1x TBE buffer. A volume of 20 µl of the PCR products was introduced into the gel. The amplicons’ size was determined using the gene a DNA ladder (peqGOLD 1 kb DNA-Ladder, Peqlab, Germany). Electrophoresis was performed at a voltage of 75 V and a current of 150 mA, and the gels were stained with Ethidium Bromide at a 0.5 µg/ml concentration. The resulting bands were studied under UV light. The amplicon of interest, which had an approximate length of 600 bp, was documented using a Dig-doc imaging system (UVP, INC, England) using Totallab software.

### 18S RNA sequencing and phylogenetic relation

PCR results were purified with the help of a Gene JET PCR Purification Kit (Thermo Scientific) with silica-based micro spin columns. Macrogen Inc., Korea, performed the product sequencing reaction in one direction with an ABI PRISM 3100 Genetic Analyzer (Micron-Corp. Korea). The sequences were analyzed as queries to Genbank with BLASTn http://www.ncbi.nlm.nih.gov/BLAST/) [14] to confirm the identities and closest relatives of the samples. Several sequence alignments were produced utilizing the MUSCLE algorithm, a component of the MEGA11 software (https://www.megasoftware.net/); the final sequence length was 487 bp after trimming. *Sarcocystis* spp. 18S rRNA genotypes with > 99% similarities were used to construct the phylogenetic tree (Table 1) with the Maximum Likelihood algorithm using MEGA11 software [15]. The phylogeny was evaluated through the implementation of 1000 bootstrap replications. The sequence of *Eimeria acervulina* (GenBank accession number GU593707) was utilized as an out-group.

**Table 1:** Sarcocystis species used for constructing the phylogenetic tree

Species	Source (host/country)	Accession number
*S. arieticanis*	Sheep/Spain	MK420017
*S. morae*	Red deer/Spain	KY973376
*S. tenella*	Sheep/Spain	MK420019
*S. linearis*	Roe deer/Lithuania	MN334301
*S. linearis*	Roe deer/Spain	MN334309
*S. cruzi*	Cattle/Iran	MT757928
*S. capracanis*	Sheep/Spain	MW832494
*S. tenella*	Cattle/Iraq	OP302809
*S. heydorni*	Cattle/Iran	KX057996
*S. cruzi*	Cattle/Iran	LC214880
*S. morae*	Red deer/Spain	KY973379
*S. arieticanis*	Sheep/China	MF039331
*S. tenella*	Sheep/Iraq	LC364052
*Sarcocystis* sp.	Sheep/China	MH236177
*Sarcocystis* sp.	deer meat/Japan	LC405946
*S. morae*	Fallow deer/Spain	MK790239
*S. taeniata*	Sika deer/Japan	LC481032
*S. linearis*	Roe deer/Spain	MN334313
*S. tenella*	Sheep/Iran	MT569891
*S. tenella*	Sheep/Spain	MK420018
*S. hircicanis*	Goat/China	KU820985
*S. tenella*	Sheep/Iran	KF489424

**Table 2:** The external measurements of the shark samples

External parts	Tiger shark average length/cm)	Shortfin Mako shark Length/cm
Total length	280	259
Pre-caudal length	260	230
Head length	48	53
Pre-orbital length	10	11
Pectoral fin	45	52
Dorsal fin (anterior part)	20	31
dorsal fin (posterior part)	18	5
Pelvic fin	24	7.5
Anal fin	10	6
Upper caudal fin lobe	48	39
Lower caudal fin lobe	39	36

## Results

### External and internal postmortem observation

The received sharks were identified as two male and female tiger sharks, *Galeocerdo cuvier*, Péron & Lesueur, 1822, and an adult male shortfin mako shark, *Isurus oxyrinchus*, Rafinesque, 1810. The external body measurements of the three samples are shown in Table (2).

**Fig. 2:**
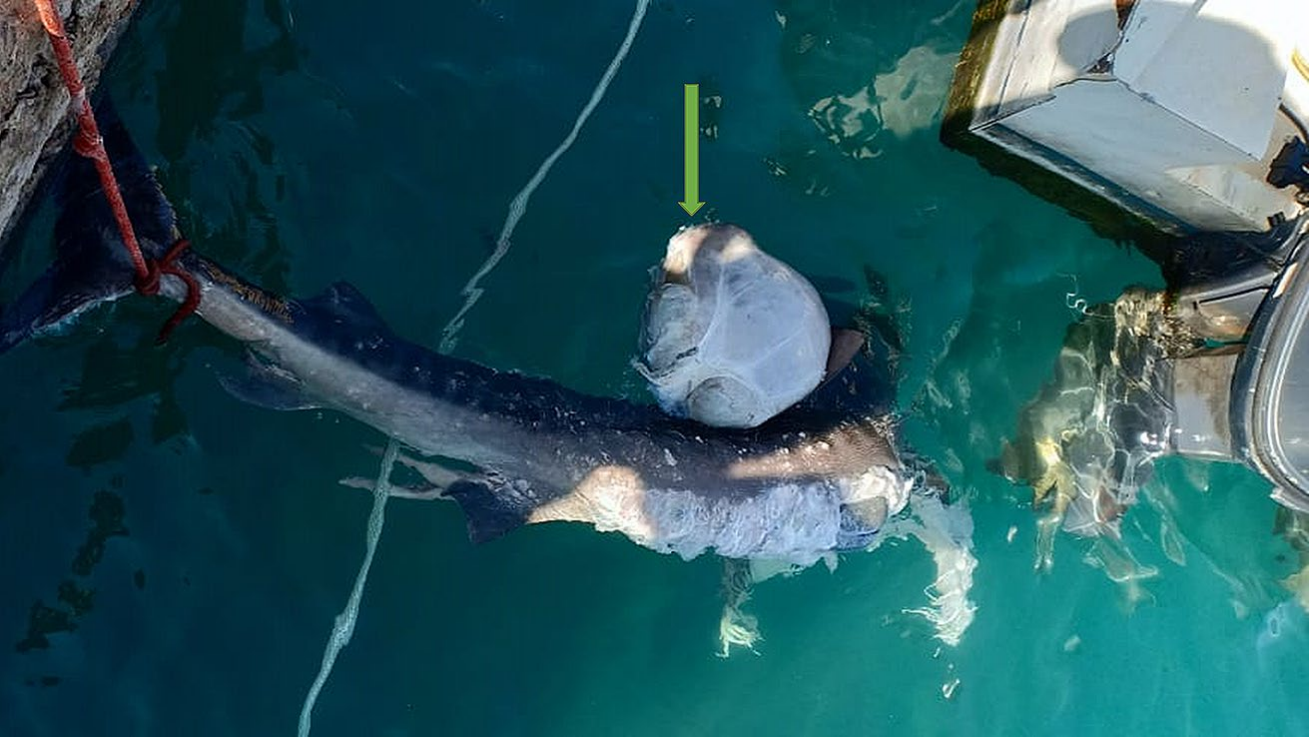
Externally situated stomach (green arrow) in a putrefied sample of the tiger shark, *Galeocerdo cuvier*

Tiger shark samples were found putrefied and judged dead several days before sampling time. One had an externally protruded stomach (Fig. 2). Opening this stomach revealed the presence of terrestrial animal remnants in the contents. At the same time, no parasitic cysts or abnormal macroscopic signs could be detected in the internal organs.

**Fig. 3:**
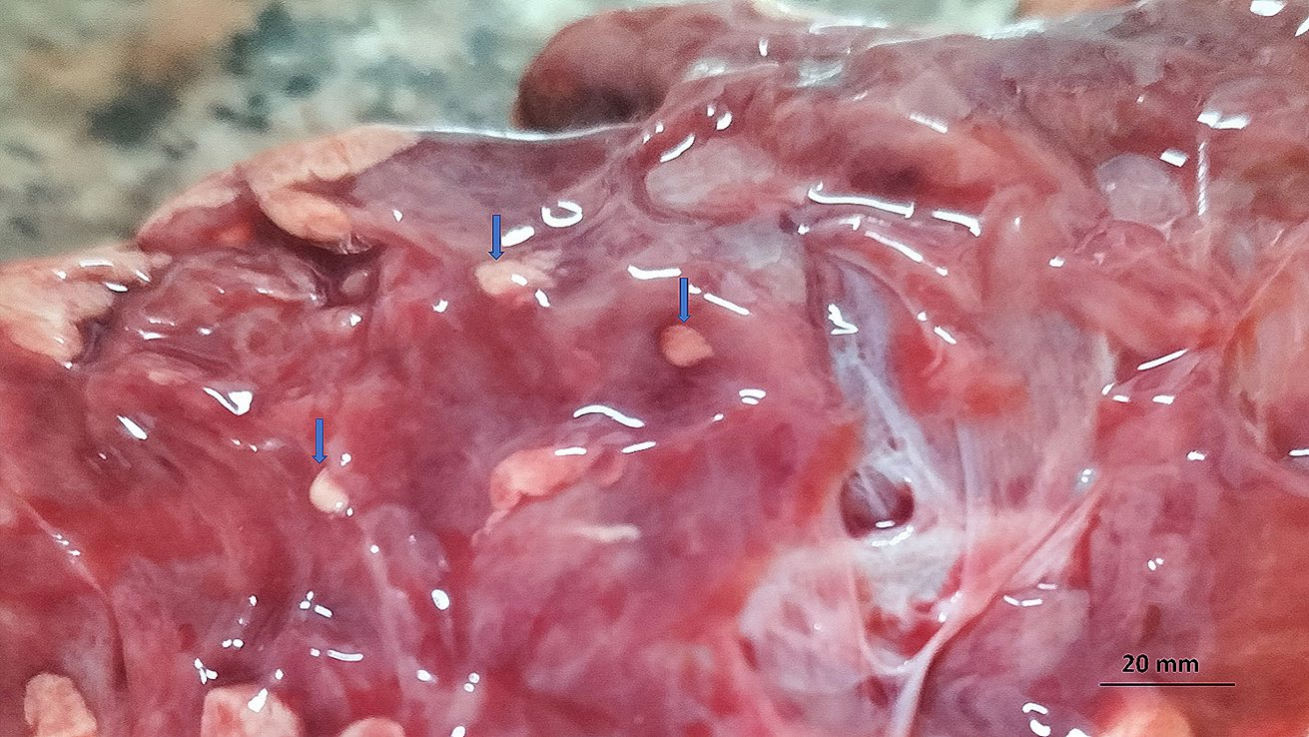
Macroscopic *Sarcocystis* (blue arrows) in the intestinal wall of mako shark, *Isurus oxyrinchus* that varies in size (from 0.5 to 1 cm) and in shape (from oval to circular)

The shortfin mako shark was lethargic and weak. The internal examination showed that the liver and the heart were pale in color, while the intestinal tract was empty of any food residues. The intestinal wall of the shortfin mako shark had whitish color cysts of different sizes and shapes, from oval to round-shaped, compatible with *Sarcocystis* cysts; their length varied from 0.5 to 1 cm (Fig. 3). no other abnormalities or parasitic cysts were detected in other organs.

### 18S RNA sequencing and phylogenetic relation

**Fig. 4:**
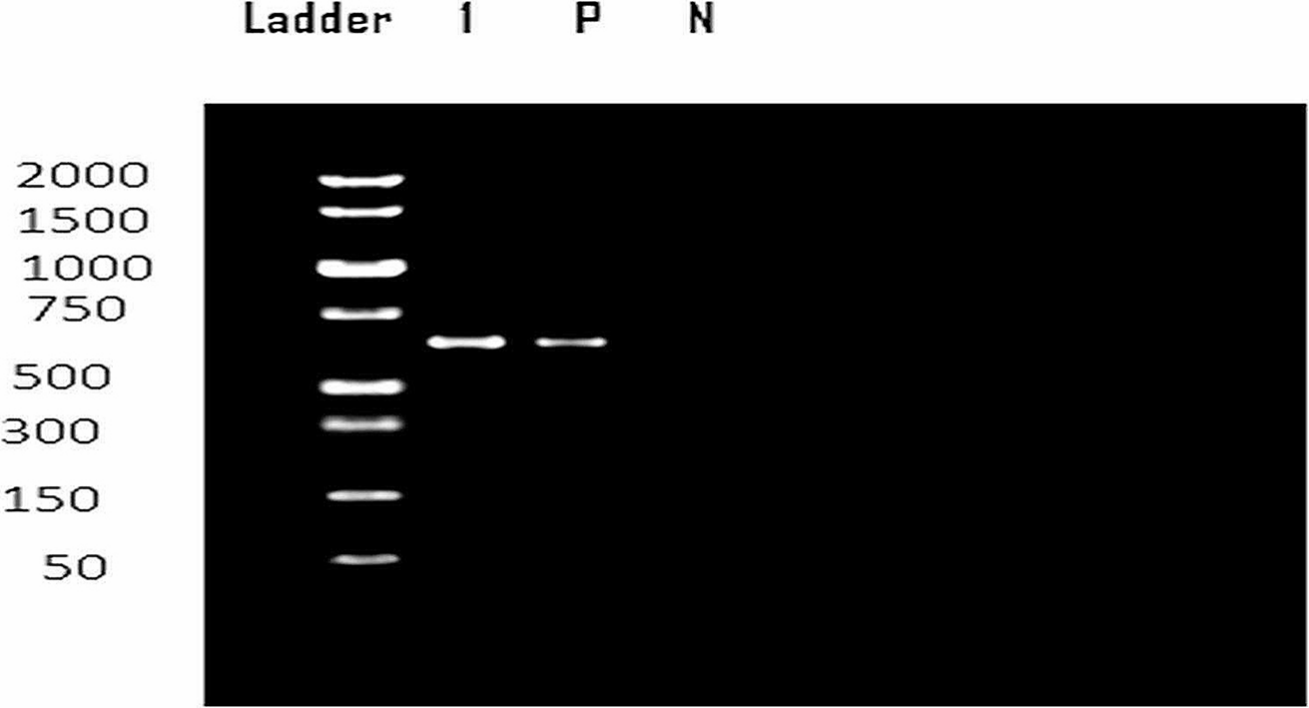
Electrophoresis of PCR product of 18S rRNA gene showing bands detected at 600 bp region belonged *Sarcocyst spp.* The figure shows the result of one representative sample. L, DNA ladder (1 kb); Lanes 1, shark samples macroscopic cyst; P, Positive control; N, negative control

PCR amplification of the small subunit 18S rRNA gene array (*Sar1* gene) of all of the samples produced a single, homogenous electrophoretic band of approximately 600 bp, as expected for a *Sarcocystis* sp. amplicon. (Fig. 4).

**Fig. 5:**
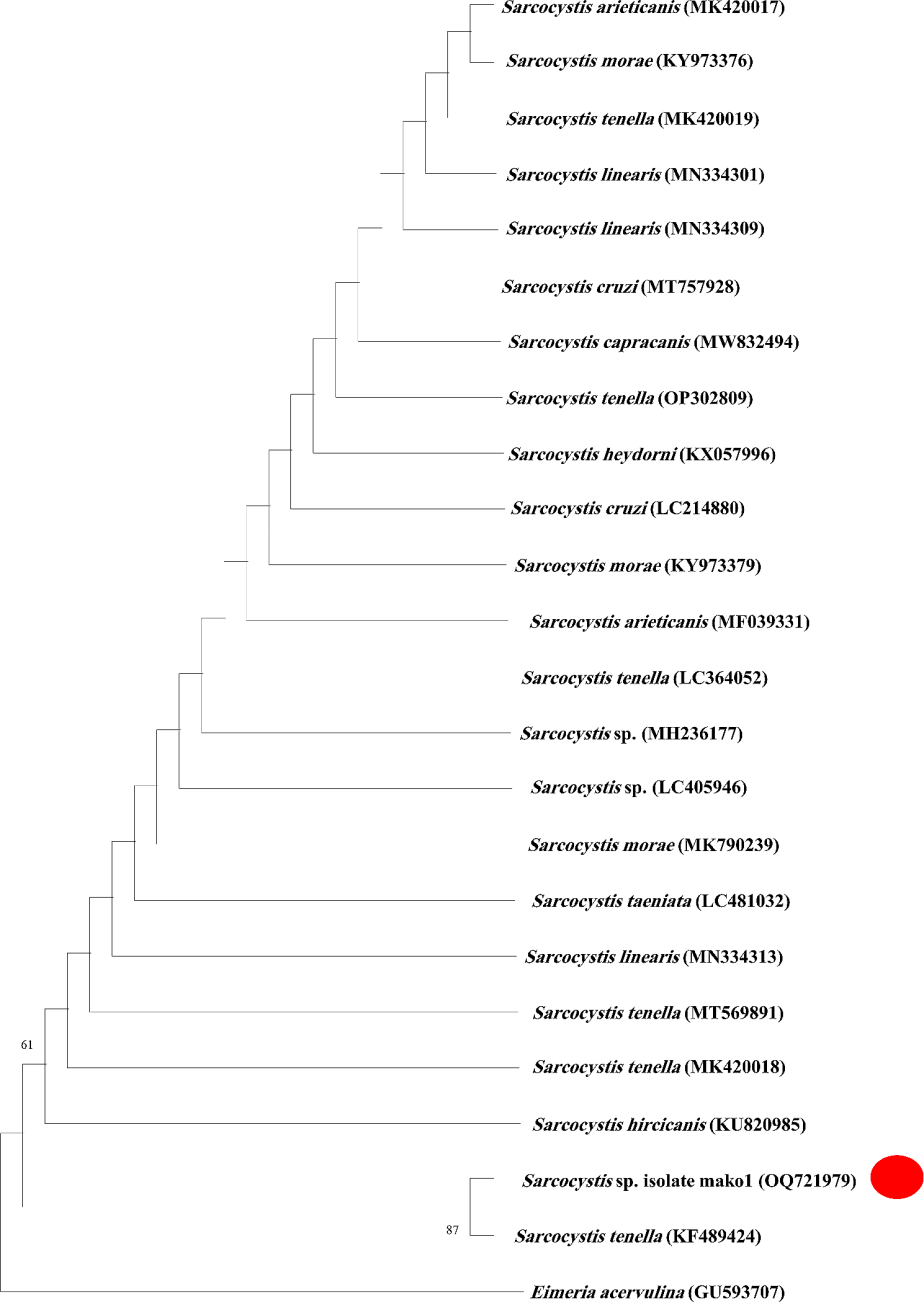
Phylogenetic tree of chosen members of the family Sarcocystidae based on 18S rDNA sequences with Eimeria acervulina as the out-group, inferred using the Maximum Likelihood Tree in MEGA software version 11. The GenBank accession numbers for every sequence that constitutes the analysis are provided before the taxon names. The sequences found in this study are shown by the red circle (GenBank accession number OQ721979). Bootstrap values that exceeded 50 were shown

The nucleotide sequence obtained from the 18S rRNA study was the same for all of the samples. It was deposited in Genbank (www.ncbi.nlm.nih.gov/genbank) with the accession number OQ721979. This sequence exhibited a complete nucleotide sequence homology of 100% with the reference isolate of *S. tenella* from sheep in Iran (GenBank accession number KF489424), and it constitutes the first record of *Sarcocystis* sp. in mako shark in the Red Sea. It presents a high identity with other *Sarcocystis* spp., as described in the phylogenetic tree (Fig. 5).

## Discussion

Shark sampling in the Red Sea poses challenges due to the restricted measures imposed in these protected areas by Egyptian environmental laws 4-1994 and 1095–2011, which prohibit fishing of cartilaginous fish [16, 17], along with listing the shortfin mako sharks as endangered species [18], and the Tiger sharks as near-threatened species [19] in the IUCN Red List of Threatened Species. The limited number of samples under current investigation is because of the constraints above.

Multiple species of *Sarcocystis* have been recorded in marine mammals. In 1998 and 1999, *Sarcocystis* sp. was reported in the cardiac and skeletal muscles of Atlantic white-sided dolphins, *Largenorhynchus acutus* from Massachusetts, North America [10]. In 2002, *S. canis* was reported to be the causative agent of fatal hepatic infections in *Stenella coeralealba* dolphins in Spain [20], *Sarcocystis neurona* was registered as the causative agent of encephalitis in both captive [21] and wild sea otters [22–24], in addition to causing myositis in sea lion, *Zalophus californianus* [25]. Beluga whales and an Atlantic white-sided dolphin are also proposed as intermediate hosts for *Sarcocystis* sp., located in their striated muscles [9]. In this study, *Sarcocystis* sp. was recorded in the intestinal wall of the Red Sea shortfin mako shark, *Isurus oxyrinchus*, which, to our knowledge, is the first documented case in a fish.

The PCR method is considered a valuable tool for identifying *Sarcocystis* alongside fundamental morphological methods to overcome the difficulties and erroneous results of the later [26]. Together with the gross examination, the 18S rRNA gene screening in the current study confirmed the identification of *Sarcocystis* sp. in the shortfin mako shark, showing 100% identity with *S. tenella* from sheep (Iran: KF489424) and high identity (99.18%) with other species of Sarcocystis. The high identity between the *Sarcocystis* sequence obtained in this study and another 22 *Sarcocystis* spp. sequences was confirmed through the phylogenetic analyses where *Sarcocystis* from mako shark (OQ721979) clustered as a sister taxa with *S. tenella* isolated from Irani sheep (KF489424). The 100% pairwise identity of the genotype from the intestinal wall of the shortfin mako shark and the sheep muscle genotype suggests the occurrence of the same species in the Red Sea waters and highlights an uncommon path for the closing of Sactocystis life cycles in the marine environment.

**Fig. 6:**
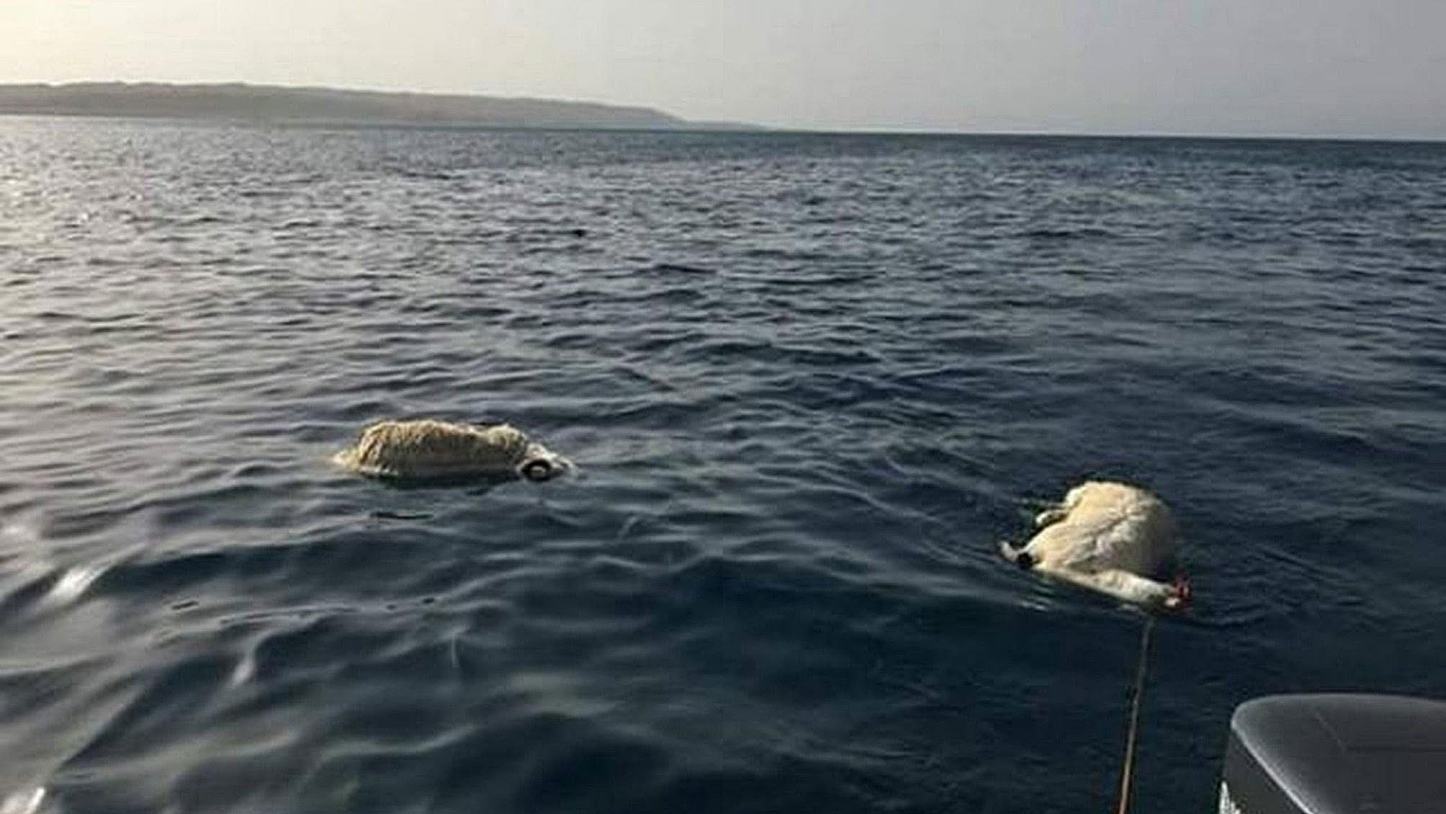
Reporting of animal carcasses found in the Red Sea, 2018. Original photo [29]

The transmission of *Sarcosystis* in marine mammals was suggested to be either from terrestrial sources that may contaminate the marine water [21, 27], the invertebrate hosts such as marine mussels that can ingest parasites and act as a source for the infection [28], or the marine mammals which may act as an intermediate host for the infection [10]. In an unpublished report by the Red Sea Marine Park Authority (RSMPA), it has been reported that in 2018, a large number of terrestrial animal carcasses were thrown from livestock carriers into the water of the Red Sea [29] (Fig. 6). Furthermore, in the current study, terrestrial animal remnants were found in the stomach of tiger shark samples indicating that the above case is not an isolated practice. Both findings support that the presence of *Sarcocystis* sp. in the shortfin mako shark, *Isurus oxyrinchus*, is likely due to the direct contact between the terrestrial animal and the marine environment, either by disposal of whole animals or of animal excretions from livestock carriers. A previous study recorded the occurrence of several Sarcocystis species, such as *S. tenella*, in water samples from Lithuania [30]. However, reports of the involvement of fish hosts in *Sarcocystis* spp. life cycles are quite scarce and poorly documented.

*Sarcocystis* is typically a host-specific parasite requiring a 2-host cycle [2]. The existence of *Sarcocystis* macroscopic cysts in the intestinal walls of shortfin mako shark strongly suggests that this fish, and likely other fish species, can act as intermediate hosts for *Sarcocystis* genotypes, including livestock species of public safety concern. The existence of *Sarcocystis* in the shortfin Mako shark put this shark on the list of intermediate hosts. The definitive hosts may be the higher marine animals that feed on dead infected sharks in the same ecosystem as larger sharks and Great white sharks, *Carcharodon carcharias* [31]. The completion of *Sarcocystis* spp. cycles in aquatic ecosystems has obvious environmental and safety implications and deserves further investigation.

## Conclusion

In conclusion, our results exhibit molecular confirmation to incorporate fish as potential intermediate hosts of *Sarcocystis* sp. infection, as it has been recorded as macroscopic cysts in the intestine of a shortfin mako shark collected from Red Sea water via 18S rRNA gene amplification and sequencing. More updates are required to recognize these infections’ full life cycle and pathological effects on aquatic hosts. Further research should also focus on using other gene markers to track the epidemiology and taxonomy status of *Sarcocystis infection*s in Egyptian marine ecosystems. Furthermore, this study highlights an unexpected relationship between the disposal of dead animals or animal wastes by livestock carriers in the Red Sea waters. A binding strategy must be developed to prevent these practices.

### Electronic supplementary material

Below is the link to the electronic supplementary material.

Supplementary Material 1

Supplementary Material 2

Supplementary Material 3

Supplementary Material 4

Supplementary Material 5

Supplementary Material 6

Supplementary Material 7

## Data Availability

The sequence obtained in the study has been uploaded to the GeneBank database under the accession number OQ721979.

## Abbreviations

PCR: The polymerase chain reaction
rRNA: ribosomal RNA
dNTP: deoxyribonucleotide triphosphate

## References

[1] Saeed MA, Rashid MH, Vaughan J, Jabbar A (2018). Sarcocystosis in South American camelids: the state of play revisited. Parasites Vectors.

[2] Dubey JP, Calero-Bernal R, Rosenthal BM, Speer CA, Fayer R. Sarcocystosis of animals and humans, 2nd edition. CRC Press, Boca Raton. 2016; pp 195–214 217–234, 243–248, 273–275.

[3] Spickler AR, Sarcocystosis. August. Available online: http://www.cfsph.iastate.edu/Factsheets/pdfs/sarcocystosis.pdf. (accessed on 21 2020).

[4] Shams M, Shamsi L, Asghari A, Motazedian MH, Mohammadi-Ghalehbin B, Omidian M, Nazari N, Sadrebazzaz A (2022). Molecular epidemiology, species distribution, and zoonotic importance of the neglected meat-borne pathogen *Sarcocystis* spp. in cattle (Bos taurus): a global systematic review and meta-analysis. Acta Parasitol.

[5] Dubey JP, Hilali M, Van Wilpe E, Calero-Bernal R, Verma SK, Abbas IE (2015). A review of sarcocystosis in camels and redescription of *Sarcocystis Cameli* and *Sarcocystis Ippeni* sarcocysts from the one-humped camel (Camelus dromedarius). Parasitology.

[6] Dubey JP, Speer CA, Fayer R. Sarcocystosis of animals and man. CRC Press, Inc.; 1989.

[7] Lapointe JM, Duignan PJ, Marsh AE, Gulland FM, Barr BC, Naydan DK, King DP, Farman CA, Huntingdon KA, Lowenstine LJ. Meningoencephalitis due to a *Sarcocystis neurona*-like protozoan in Pacific harbor seals (*Phoca vitulina richardsi*). J Parasitol. 1998 Dec;1:1184–9. 9920311

[8] Barbosa L, Johnson CK, Lambourn DM, Gibson AK, Haman KH, Huggins JL, Sweeny AR, Sundar N, Raverty SA, Grigg ME (2015). A novel *Sarcocystis neurona* genotype XIII is associated with severe encephalitis in an unexpectedly broad range of marine mammals from the northeastern Pacific Ocean. Int J Parasitol.

[9] De Guise S, Lagacé A, Girard C, Béland P (1993). Intramuscular *Sarcocystis* in two beluga whales and an Atlantic white-sided dolphin from the St. Lawrence Estuary, Quebec, Canada. J Vet Diagn Invest.

[10] Ewing R, Zaias J, Stamper MA, Bossart GD, Dubey JP (2002). Prevalence of *Sarcocystis* sp. in stranded Atlantic white-sided dolphins (*Lagenorhynchus acutus*). J Wildl Dis.

[11] Thomas NJ, Dubey JP, Lindsay DS, Cole RA, Meteyer CU (2007). Protozoal meningoencephalitis in sea otters (Enhydra lutris): a histopathological and immunohistochemical study of naturally occurring cases. J Comp Pathol.

[12] Wendte JM, Miller MA, Lambourn DM, Magargal SL, Jessup DA, Grigg ME (2010). Self-mating in the definitive host potentiates clonal outbreaks of the apicomplexan parasites *Sarcocystis neurona* and *Toxoplasma Gondii*. PLoS Genet.

[13] Bahari P, Salehi M, Seyedabadi M, Mohammadi A (2014). Molecular identification of macroscopic and microscopic cysts of *Sarcocystis* in sheep in North Khorasan Province, Iran. Int J Mol Cell Med.

[14] Altschul S, Gish W, Miller W, Myers E, Lipman D. Basic Local Alignment Search Tool. Journal of molecular biology 215, 403–410, doi: 10.1016. S0022-2836 (05). 1990:80360-2. 10.1016/S0022-2836(05)80360-2 2231712

[15] Tamura K, Stecher G, Kumar S (2021). MEGA11: molecular evolutionary genetics analysis version 11. Mol Biol Evol.

[16] Egyptian Environmental Law 4. 1994. https://www.eeaa.gov.eg/Laws/55/index.

[17] Egyptian Environmental Law 1095. 2011. https://www.eeaa.gov.eg/Laws/55/index.

[18] Rigby CL, Barreto R, Carlson J, Fernando D, Fordham S, Francis MP, Jabado RW, Liu KM, Marshall A, Pacoureau N, Romanov E. Isurus oxyrinchus, The IUCN Redlist of Threatened Species 2019.

[19] Ferreira LC, Simpfendorfer C. Galeocerdo cuvier. The IUCN Red List of Threatened Species 2019. 10.2305/IUCN.UK.2019-1.RLTS.T39378A2913541.en.

[20] Resendes AR, Juan-Sallés C, Almeria S, Majo N, Domingo M, Dubey JP (2002). Hepatic sarcocystosis in a striped dolphin (*Stenella coeruleoalba*) from the Spanish Mediterranean coast. J Parasitol.

[21] Rosonke BJ, Brown SR, Tornquist SJ, Snyder SP, Garner MM, Blythe LL (1999). Encephalomyelitis associated with a *Sarcocystis neurona*-like organism in a sea otter. J Am Vet Med Assoc.

[22] Lindsay DS, Thomas NJ, Dubey JP (2000). Biological characterization of *Sarcocystis neurona* isolated from a Southern sea otter (*Enhydra lutris nereis*). Int J Parasitol.

[23] Lindsay DS, Thomas NJ, Rosypal AC, Dubey JP (2001). Dual *Sarcocystis neurona* and *Toxoplasma Gondii* infection in a Northern Sea Otter from Washington State, USA. Vet Parasitol.

[24] Miller MA, Crosbie PR, Sverlow K, Hanni K, Barr BC, Kock N, Murray MJ, Lowenstine LJ, Conrad PA (2001). Isolation and characterization of *Sarcocystis* from brain tissue of a free-living southern sea otter (*Enhydra lutris nereis*) with fatal meningoencephalitis. Parasitol Res.

[25] Carlson-Bremer DP, Gulland FM, Johnson CK, Colegrove KM, Van Bonn WG (2012). Diagnosis and treatment of *Sarcocystis neurona*–induced myositis in a free-ranging California sea lion. J Am Vet Med Assoc.

[26] Kamber U, Arslan MÖ, Gülbaz G, Taşçi GT, Akca A (2018). Identification of *Sarcocystis* spp. by polymerase chain reaction and microscopic examination in various beef products (minced meat, meatballs, fermented sausage). Turkish J Veterinary Anim Sci.

[27] O’Byrne AM, Lambourn DM, Rejmanek D, Haman K, O’Byrne M, VanWormer E, Shapiro K (2021). *Sarcocystis Neurona* transmission from opossums to marine mammals in the Pacific Northwest. EcoHealth.

[28] Michaels L, Rejmanek D, Aguilar B, Conrad P, Shapiro K (2016). California mussels (*Mytilus californianus*) as sentinels for marine contamination with *Sarcocystis Neurona*. Parasitology.

[29] Ghallab A. Findings of animal carcasses in the Red Sea water. Internal report of the Red Sea Marine Park Authority (RSMPA). 2018 *Unpublished report*.

[30] Strazdaitė-Žielienė Ž, Baranauskaitė A, Butkauskas D, Servienė E, Prakas P (2022). Molecular Identification of Parasitic Protozoa *Sarcocystis* in Water samples. Veterinary Sci.

[31] Fergusson IK, Compagno LJ, Marks MA (2000). Predation by white sharks *Carcharodon carcharias* (Chondrichthyes: Lamnidae) upon chelonians, with new records from the Mediterranean Sea and a first record of the ocean sunfish *Mola mola* (Osteichthyes: Molidae) as stomach contents. Environ Biol Fish.

