# Supplementary figures and images for: First molecular identification and phylogenetic illustration of Sarcocystis species infection in Red Sea shortfin mako shark (Isurus oxyrinchus Rafinesque, 1810)
Source: BMC Vet Res. 2024 Mar 15;20:104. doi: 10.1186/s12917-024-03952-w (PMC10941371; doi:10.1186/s12917-024-03952-w)

**
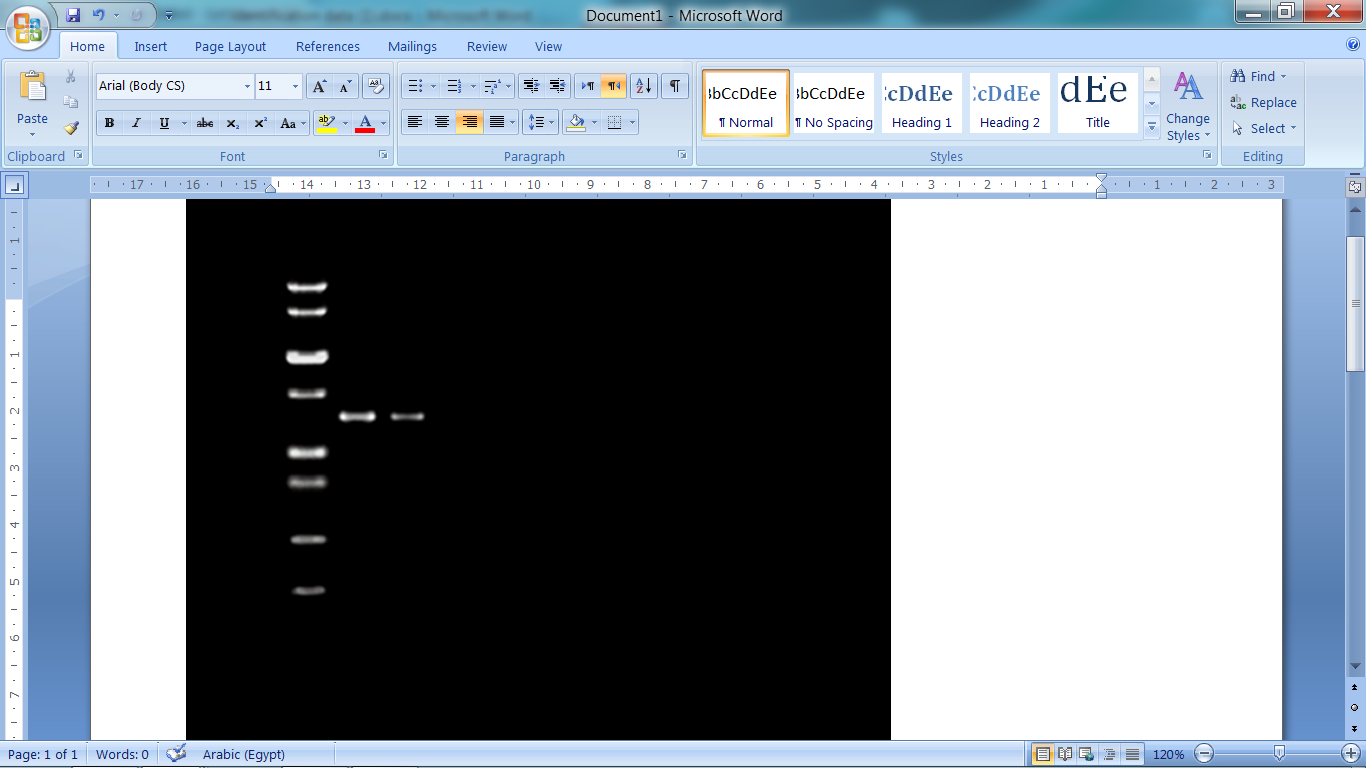
Supplementary file 1: Figure S1.** Specific genomic product for Sarcocystis sample with ≈ 600 bp.

Supplement: Supplementary file 1 — Supplementary Material 1 [file 12917_2024_3952_MOESM1_ESM.docx]

**Supplementary file 4: Figure S4.** Dendrogram of DNA Ladder sample.


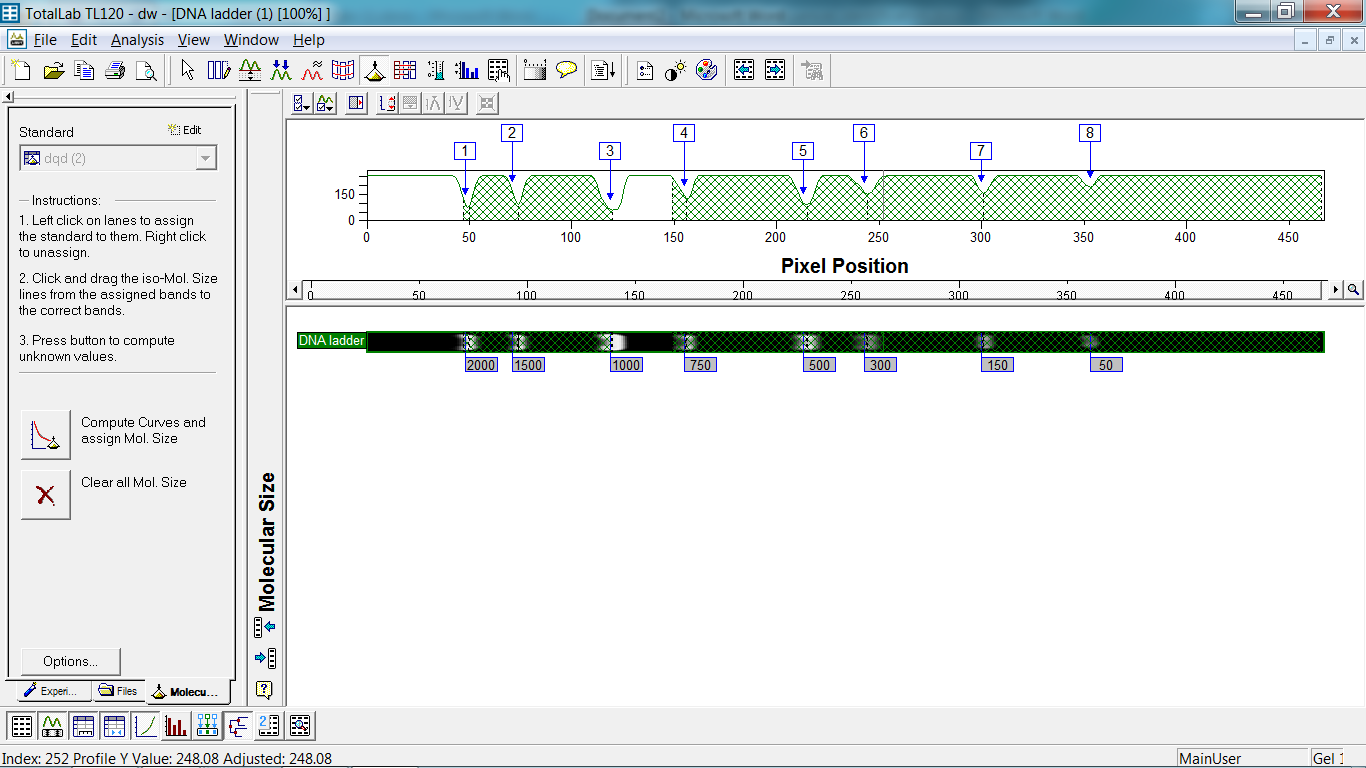

Supplement: Supplementary file 4 — Supplementary Material 4 [file 12917_2024_3952_MOESM4_ESM.docx]

**Supplementary file 5: Figure S5.** Dendrogram of Sarcocystis sample with ≈ 600 bp.


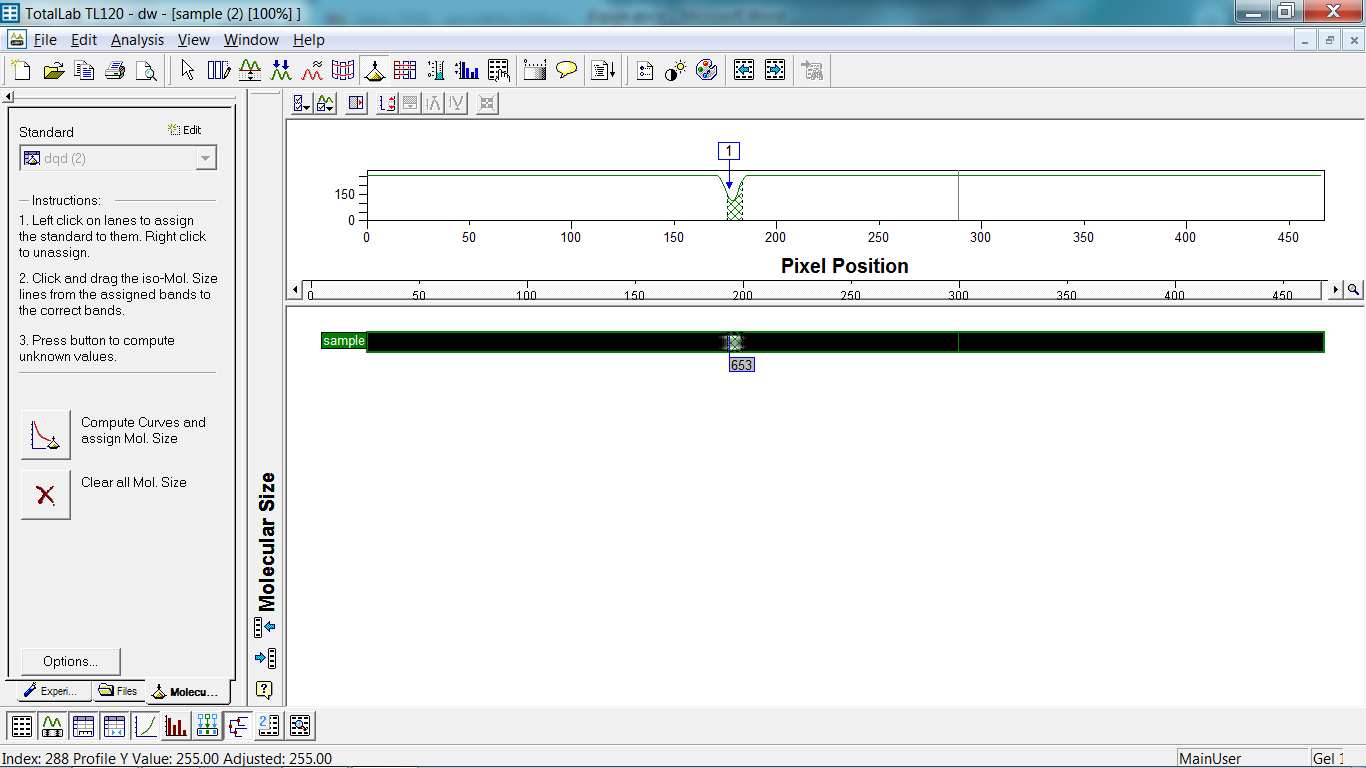

Supplement: Supplementary file 5 — Supplementary Material 5 [file 12917_2024_3952_MOESM5_ESM.docx]

**Supplementary file 6: Figure S6.** Dendrogram of the positive control sample.


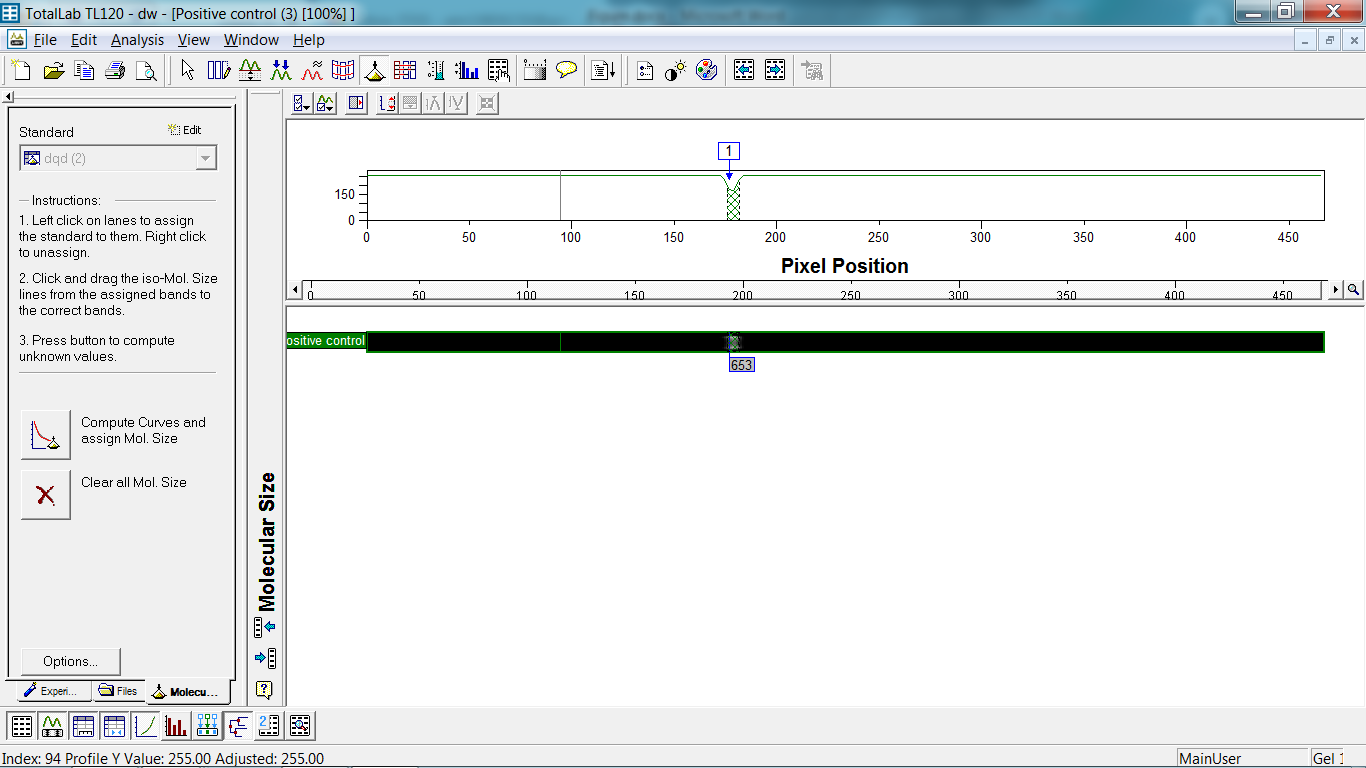

Supplement: Supplementary file 6 — Supplementary Material 6 [file 12917_2024_3952_MOESM6_ESM.docx]

# Supplementary file 7: Figure S7.
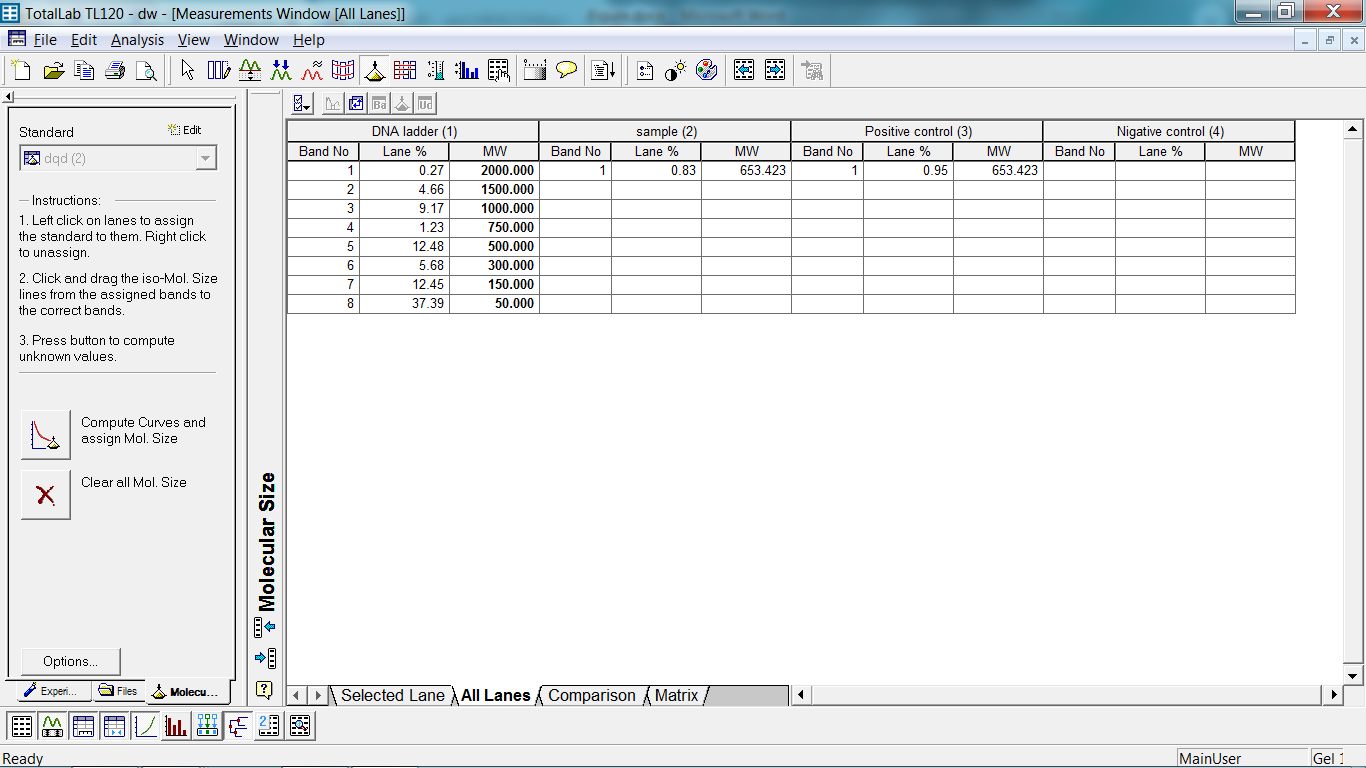
Data parameters for DNA ladder and *Sarcocystis* sample with ≈ 600 bp.

Supplement: Supplementary file 7 — Supplementary Material 7 [file 12917_2024_3952_MOESM7_ESM.docx]
