# Supplementary material for: First molecular identification and phylogenetic illustration of Sarcocystis species infection in Red Sea shortfin mako shark (Isurus oxyrinchus Rafinesque, 1810)
Source: BMC Vet Res. 2024 Mar 15;20:104. doi: 10.1186/s12917-024-03952-w (PMC10941371; doi:10.1186/s12917-024-03952-w)
Supplement: Supplementary file 2 — Supplementary Material 2 [file 12917_2024_3952_MOESM2_ESM.docx]

**
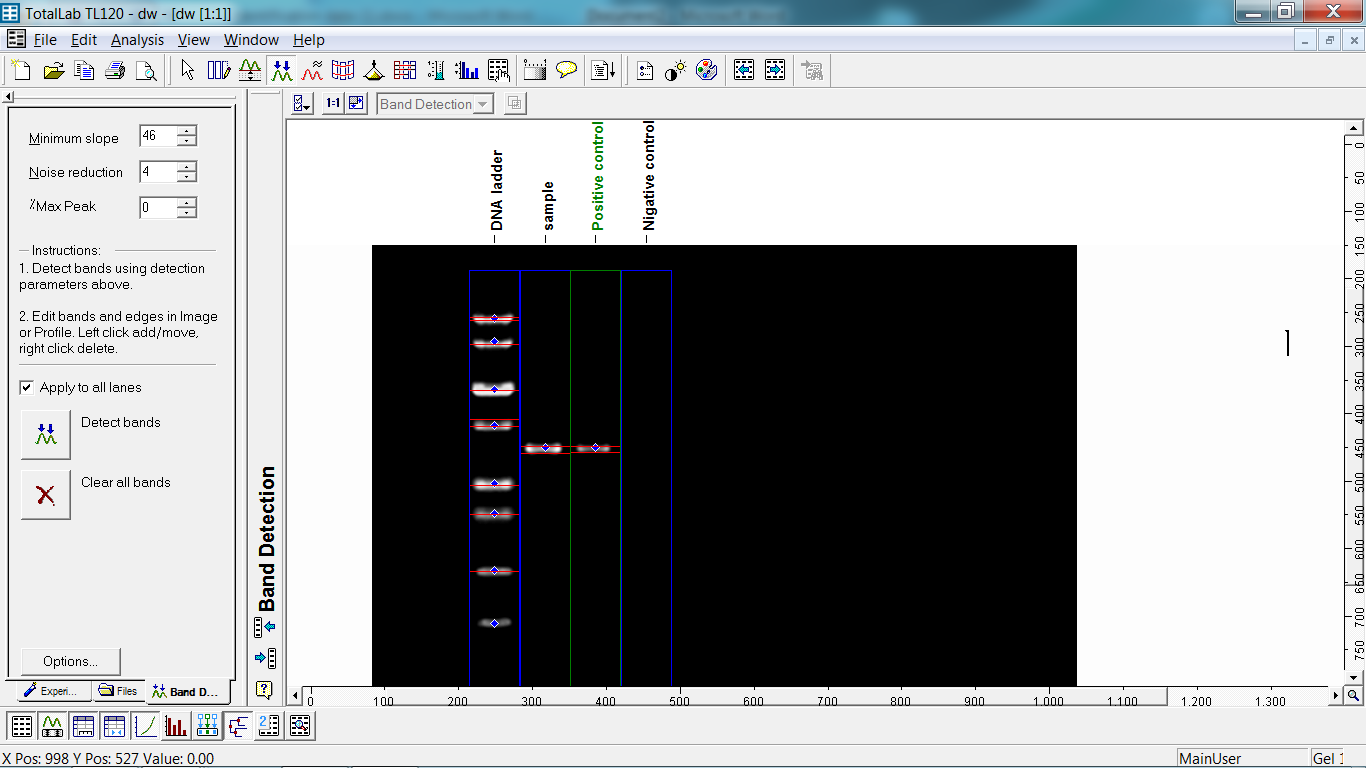
Supplementary file 2: Figure S2.** Computerized detection of specific genomic product for Sarcocystis sample with ≈ 600 bp.
